# Hybrid epicardial-endocardial ablation for long-standing persistent atrial fibrillation: A subanalysis of the CONVERGE Trial

**DOI:** 10.1016/j.hroo.2022.11.007

**Published:** 2022-12-05

**Authors:** David B. DeLurgio, Christopher Blauth, Michael E. Halkos, Karl J. Crossen, David Talton, Saumil R. Oza, Anthony R. Magnano, Mark A. Mostovych, Sreedhar Billakanty, Steven Duff, Christopher Stees, Jason Sperling, Syed Ahsan, John Yap, Christian Shults, David Pederson, James Garrison, Paul Tabereaux, David M. Gilligan, Graham Bundy, Otto Costantini, Eric Espinal, Angelo La Pietra, Felix Yang, Yisachar Greenberg, Israel Jacobowitz, Jaswinder Gill

**Affiliations:** ∗ St. Joseph’s Hospital, Emory University, Atlanta, Georgia; † Guy’s and St. Thomas’ Foundation Trust, London, United Kingdom; ‡ Cardiology Associates Research, LLC, Tupelo, Mississippi; § St. Vincent’s Healthcare, Jacksonville, Florida; ¶ Riverside Methodist Hospital, OhioHealth, Columbus, Ohio; || HealthOne Cardiothoracic Surgery Associates, Aurora, Colorado; ∗∗ Barts Heart Centre, St. Bartholomew’s Hospital, London, United Kingdom; †† MedStar Washington Hospital Center, Washington, DC; ‡‡ STAR Clinical Trials/Methodist Cardiology Clinic San Antonio, San Antonio, Texas; §§ Heart Center Research, LLC, Huntsville, Alabama; ¶¶ Virginia Cardiovascular Specialists, Richmond, Virginia; |||| Summa Health Medical Group, Akron, Ohio; ∗∗∗ Mount Sinai Medical Center, Miami Beach, Florida; ††† Maimonides Medical Center, Brooklyn, New York

**Keywords:** Long-standing persistent atrial fibrillation, Hybrid ablation, Epicardial, Endocardial ablation, Antiarrhythmic drug

## Abstract

**Background:**

Favorable clinical outcomes are difficult to achieve in long-standing persistent atrial fibrillation (LSPAF) with catheter ablation (CA). The CONVERGE (Convergence of Epicardial and Endocardial Ablation for the Treatment of Symptomatic Persistent Atrial FIbrillation) trial evaluated the effectiveness of hybrid convergent (HC) ablation vs endocardial CA.

**Objective:**

The study sought to evaluate the safety and effectiveness of HC vs CA in the LSPAF subgroup from the CONVERGE trial.

**Methods:**

The CONVERGE trial was a prospective, multicenter, randomized trial that enrolled 153 patients at 27 sites. A post hoc analysis was performed on LSPAF patients. The primary effectiveness was freedom from atrial arrhythmias off new or increased dose of previously failed or intolerant antiarrhythmic drugs (AADs) through 12 months. The primary safety endpoint was major adverse event incidence through 30 days with HC. Key secondary effectiveness measures included (1) percent of patients achieving ≥90% AF burden reduction vs baseline and (2) AF freedom.

**Results:**

Sixty-five patients (42.5% of total enrollment) had LSPAF; 38 in HC and 27 in CA. Primary effectiveness was 65.8% (95% confidence interval [CI] 50.7%–80.9%) with HC vs 37.0% (95% CI 5.1%–52.4%) with CA (*P =* .022). Through 18 months, these rates were 60.5% (95% CI 50.0%–76.1%) with HC vs 25.9% (95% CI 9.4%–42.5%) with CA (*P =* .006). Secondary effectiveness rates were higher than CA with HC at 12 and 18 months. Freedom from atrial arrhythmias off AADs was 52.6% (95% CI 36.8%–68.5%) and 47.4% (95% CI 31.5%–63.2%) with HC at 12 and 18 months vs 25.9% (95% CI 9.4%–42.5%) and 22.2% (95% CI 6.5%–37.9%) with CA, respectively (12 months: *P =* .031; 18 months: *P =* .038). Three (7.9%) major adverse events occurred within 30 days of HC.

**Conclusion:**

Post hoc analysis demonstrated effectiveness and acceptable safety of HC compared with CA in LSPAF.

**Key Findings**

▪ Hybrid convergent ablation is a minimally invasive approach to isolate the posterior left atrial wall and pulmonary veins using combined epicardial and endocardial ablation by a cardiac surgeon and electrophysiologist, respectively.
▪ This CONVERGE trial subanalysis of patients with long-standing persistent atrial fibrillation (AF) showed that the rates of freedom from atrial arrhythmias off a new or increased dose of previously failed antiarrhythmic drugs (AADs), regardless of AADs, and off AADs were higher through 12 and 18 months after hybrid convergent ablation vs endocardial catheter ablation.
▪ A greater proportion of patients with long-standing persistent AF who received hybrid convergent ablation experienced ≥90% AF burden reduction off a new or increased dose of AADs compared with those who received catheter ablation at 12 and 18 months.
▪ Hybrid convergent ablation had improved effectiveness compared with catheter ablation in patients with long-standing persistent AF through 18 months and with reasonable safety.

## Introduction

Long-standing persistent atrial fibrillation (LSPAF) is characterized by the presence of continuous persistent atrial fibrillation (AF) for >1 year.^1^ As the duration of persistent AF increases, progressive electroanatomical remodeling occurs that perpetuates AF further, often summarized by the statement “AF begets AF.” AF triggers and substrate extend beyond the pulmonary veins (PVs) and into the left atrial posterior wall, left atrial appendage, and other sites.^2^ Therefore, it is not unexpected that PV isolation (PVI)–only ablation is not as clinically successful in persistent AF and LSPAF as it is in paroxysmal AF. Despite evidence that regions outside of the PVs are important in initiating and sustaining persistent AF, endocardial catheter ablation of these regions for persistent AF and LSPAF has yielded inconsistent and even suboptimal clinical outcomes, perhaps most notably the STAR-AF II (Substrate and Trigger Ablation for Reduction of Atrial Fibrillation) trial.^3^ In a systematic review of observational and randomized endocardial catheter ablation studies for LSPAF, Brooks and colleagues^4^ reported a range of single procedure success rates with antiarrhythmic drug (AADs) from 21% to 68%. These rates stemmed from various endocardial ablation approaches from PVI only to PVI plus additional linear ablation, posterior wall box, or complex fractionated atrial electrograms, and stepwise ablation. On the other end of the spectrum, Cox-Maze IV surgical ablation has shown favorable efficacy in patient populations with predominantly persistent AF and LSPAF.^5^^,^^6^ Long-term efficacy of Cox-Maze IV as a stand-alone procedure for LSPAF was recently reported^7^; however, most Cox-Maze IV procedures are performed in conjunction with planned open heart surgical procedures, thus limiting the applicable LSPAF patient population. Taken together, there is a clinical gap in effective treatment options for most patients with LSPAF.

Hybrid epicardial-endocardial convergent ablation was developed in 2009 as a multidisciplinary, stand-alone ablation procedure performed by cardiothoracic surgery and electrophysiology.^8^ The goal is to create a transmural lesion set that achieves durable PV and left atrial posterior wall isolation. The prospective, multicenter randomized CONVERGE trial evaluated safety of the hybrid convergent procedure and compared its effectiveness with endocardial catheter ablation for persistent or LSPAF. ^9^ Minimally invasive surgical epicardial ablation of the left atrial posterior wall was performed with a unipolar radiofrequency (RF) catheter followed by endocardial RF catheter ablation to complete PVI and address gaps to ensure posterior wall isolation. This hybrid ablation strategy was compared with a conventional endocardial RF catheter ablation strategy including PVI and roof line. The CONVERGE trial met its primary effectiveness endpoint, showing superior freedom from atrial arrhythmias off a new or increased dose previously failed AADs with hybrid convergent ablation (67.7%) compared with endocardial ablation (50%) at 12 months (*P* = .036), with a 30-day major adverse event rate (MAE) (7.8%) that also met the primary safety endpoint.

To explore the impact of hybrid convergent ablation on LSPAF, for which there is a lack of effective treatments, we performed a post hoc analysis of the CONVERGE trial focused on the LSPAF subpopulation.

## Methods

### Study design

The CONVERGE trial was a prospective, multicenter, 2:1 randomized investigational device exemption clinical trial (NCT01984346).^9^^,^^10^ Patients were enrolled from 27 participating sites in the United States (n = 25) and United Kingdom (n = 2). Detailed inclusion and exclusion criteria have been previously described.^9^^,^^10^ The CONVERGE trial was conducted in accordance with the Declaration of Helsinki. Institutional review board or ethics committee approval and patient informed consent form were obtained. All enrolled patients had symptomatic, continuous AF lasting more than 7 days with no upper limit on duration of continuous AF, that was intolerant or refractory to at least 1 class I/III AADs. All patients were naïve to left atrial ablation procedures. This substudy is a post hoc analysis of patients who had LSPAF (continuous AF for >1 year duration) at the time of enrollment.

### Index procedures and lesion sets

The details of surgical (epicardial) and electrophysiology (endocardial) procedures and lesion sets have been previously published.^9^^,^^10^ In brief, in the hybrid convergent arm, pericardial access was achieved via a transdiaphragmatic or subxiphoid incision. Epicardial ablation was performed endoscopically with a unipolar RF device (EPi-Sense; AtriCure, Mason, OH) positioned inside a pericardioscopic cannula. Parallel rows of contiguous lesions were made across the posterior wall of the left atrium. Endocardial mapping was performed to identify any breakthrough locations, especially at the pericardial reflections to guide endocardial ablation. Following surgical closure, endocardial ablation was performed with an irrigated RF catheter to complete PVI, address gaps based on electroanatomical mapping, and create a cavotriscupid isthmus (CTI) line. In the catheter ablation arm, no posterior wall isolation was performed. Endocardial ablation was performed with irrigated RF for PVI, roof line, and CTI. A bidirectional block across the CTI line was confirmed, and the LA roof line was interrogated to ensure completeness of the line. Complex fractionated atrial electrogram ablations were made at the physician’s discretion.

### Follow-up

Rhythm monitoring was performed with ECG at the 7-day, 1-month, 3-month, 6-month, and 12-month in-person visits. Adverse events and medications were also reviewed at these visits. A 24-hour rhythm monitoring was performed at 6 and 12 months. A 7-day rhythm monitoring was performed at the 18-month in-person visit. Atrial fibrillation burden reduction at 12 and 18 months were compared with 24-hour rhythm monitoring performed at baseline. Scheduled rhythm monitoring in the study was via a continuously recording ambulatory adhesive patch monitor technology.

### Effectiveness and safety endpoints

The primary effectiveness and safety endpoints in this subanalysis were the same as in the full cohort analysis.^9^^,^^10^ The primary effectiveness was freedom from AF, atrial flutter (AFL), or atrial tachycardia (AT) off class I or III AADs except for a previously failed or intolerant AAD with no increase in dosage following a 3-month blanking period through 12 months postprocedure. Definitions of failure of the primary endpoint have been previously described.^10^ The primary safety was the incidence of MAEs through 30 days postprocedure in the hybrid convergent arm, with MAEs defined as cardiac tamponade, severe pulmonary stenosis (≥70% occlusion), excessive bleeding requiring transfusion or >20% drop in hematocrit, myocardial infarction, stroke, transient ischemic attack, phrenic nerve injury, and death.^1^

Secondary effectiveness endpoints were achievement of 90% reduction of AF burden off all class I or III AADs from baseline to 12 months postprocedure except for a previously failed or intolerant AAD with no increase in dosage following a 3-month blanking period through 12 months postprocedure; achievement of 90% reduction of AF burden regardless of class I or III AAD status from baseline to 12 months postprocedure; freedom from AF off all class I or III AADs except for a previously failed or intolerant AAD with no increase in dosage following a 3-month blanking period through 12 months postprocedure; and freedom from AF regardless of class I or III AAD status following a 3-month blanking period through 12 months postprocedure. The exploratory effectiveness endpoint was achievement of 90% AF burden reduction with and without AADs from baseline to 18 months postprocedure. Additional endpoints were based on Heart Rhythm Society consensus statement alternative definitions of treatment success: freedom from atrial arrhythmias after the 3-month blanking period through 12 and 18 months off AADs and regardless of AAD status. ^1^

### Statistical considerations

All endpoints are reported in the intention-to-treat population. In the LSPAF subgroup, there were no missing arrhythmia data; missing AF burden data were not imputed. All analyses in the LSPAF subgroup were post hoc and should be interpreted with caution because confidence intervals (CIs) and *P* values were not adjusted for multiplicity. *P* values were computed using chi-square test, Fisher’s exact test, or a 2-sample *t* test, where appropriate. CIs were computed using the Wald asymptotic method or Clopper-Pearson (exact) method. AF burden reduction off a new or increased dose of previously failed or intolerant AADs at 12 months from baseline and freedom from AF off a new or increased dose of previously failed or intolerant AADs through 12 months were evaluated in a prespecified fixed sequence testing procedure. Burden was calculated as time in AF over total monitoring time. Statistical analysis was provided by NAMSA (Northwood, OH) using SAS version 9.4 (SAS Institute, Cary, NC).

## Results

### Baseline and procedural characteristics

A total of 65 (42.5%) out of the 153 patients randomized and treated in the CONVERGE trial had symptomatic, drug-refractory LSPAF preoperatively; 38 patients were in the hybrid convergent arm and 27 were in the catheter ablation arm. Baseline characteristics for these patients are shown in Table 1. Mean time since persistent AF diagnosis in the LSPAF patients was 6.0 years in the hybrid convergent arm and 5.9 years in the catheter ablation arm.

**Table 1:** Baseline characteristics of long-standing persistent atrial fibrillation subgroup

Parameter	Hybrid convergent (n = 38)	Catheter ablation (n = 27)	*P* value
Age, y	61.5 ± 10.27	65.2 ± 7.26	.280
Male	82 (31/38)	56 (15/27)	.023
Caucasian	95 (36/38)	96 (26/27)	1.000
Height, cm	178.5 ± 9.05	172.7 ± 8.95	.013
Weight, kg	104.3 ± 20.56	105.3 ± 23.10	.857
Body mass index, kg/m^2^	32.7 ± 5.97	35.4 ± 7.88	.126
Years since persistent AF diagnosis	6.0 ± 6.4	5.8 ± 5.5	.714
Left atrial diameter, cm	4.5 ± 0.68	4.3 ± 0.56	.273
Left ventricular ejection fraction, %	54.4 ± 7.50	54.7 ± 6.22	.814

Values are mean ± SD or % (n/n). *P* values based on 2-sample *t* or Wilcoxon rank sum tests for continuous variables and chi-square or Fisher exact test, as appropriate.

AF = atrial fibrillation.

Procedural parameters are shown in Table 2. In the hybrid convergent arm, the mean total epicardial ablation time (first to last epicardial lesion) was 77.3 ± 19.9 minutes. The mean total endocardial ablation time (first to last endocardial lesion) was 139.6 ± 44.1 minutes. In the catheter ablation arm, the mean total endocardial ablation time was 180.7 ± 64.1 minutes (*P =* .006 vs hybrid convergent). A total of 5 (18.5%) of 27 patients had complex fractionated atrial electrogram ablation in the catheter ablation arm.

**Table 2:** Procedural data

Ablation procedure time	Hybrid convergent (n = 38)	Catheter ablation (n = 27)	*P* value
Total epicardial ablation time, min∗	77.3 ± 19.9	Not applicable	—
Total endocardial ablation time, min∗	139.6 ± 44.1	180.7 ± 64.1	.006

Values are mean ± SD. *P* values for hybrid convergent vs catheter ablation calculated using 2-sample *t* test.

∗ Time from first lesion to last lesion.

### Twelve-month outcomes in LSPAF

**Figure 1:**
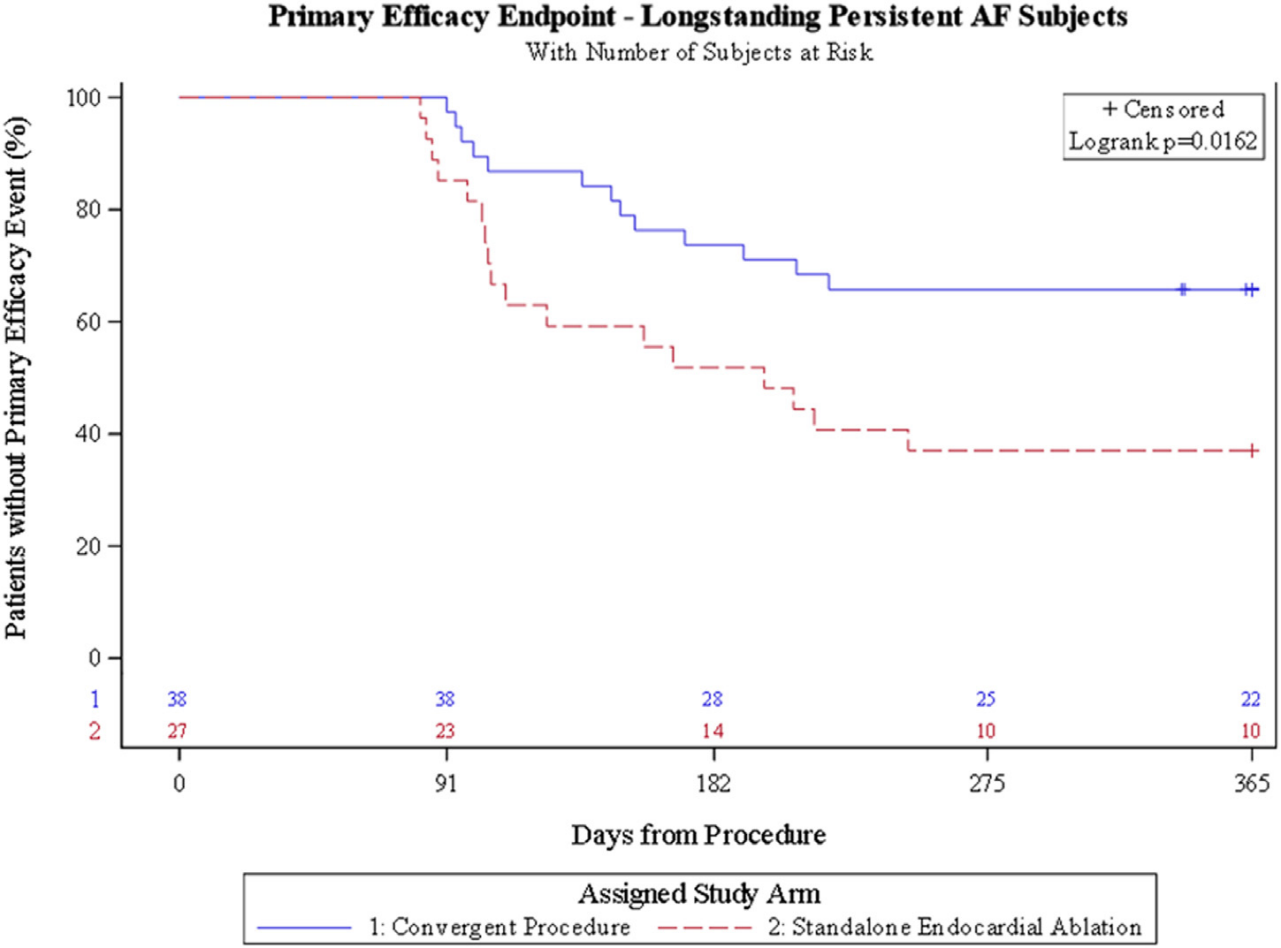
Kaplan-Meier analysis of freedom from atrial fibrillation (AF), atrial flutter, or atrial tachycardia.

**Figure 2:**
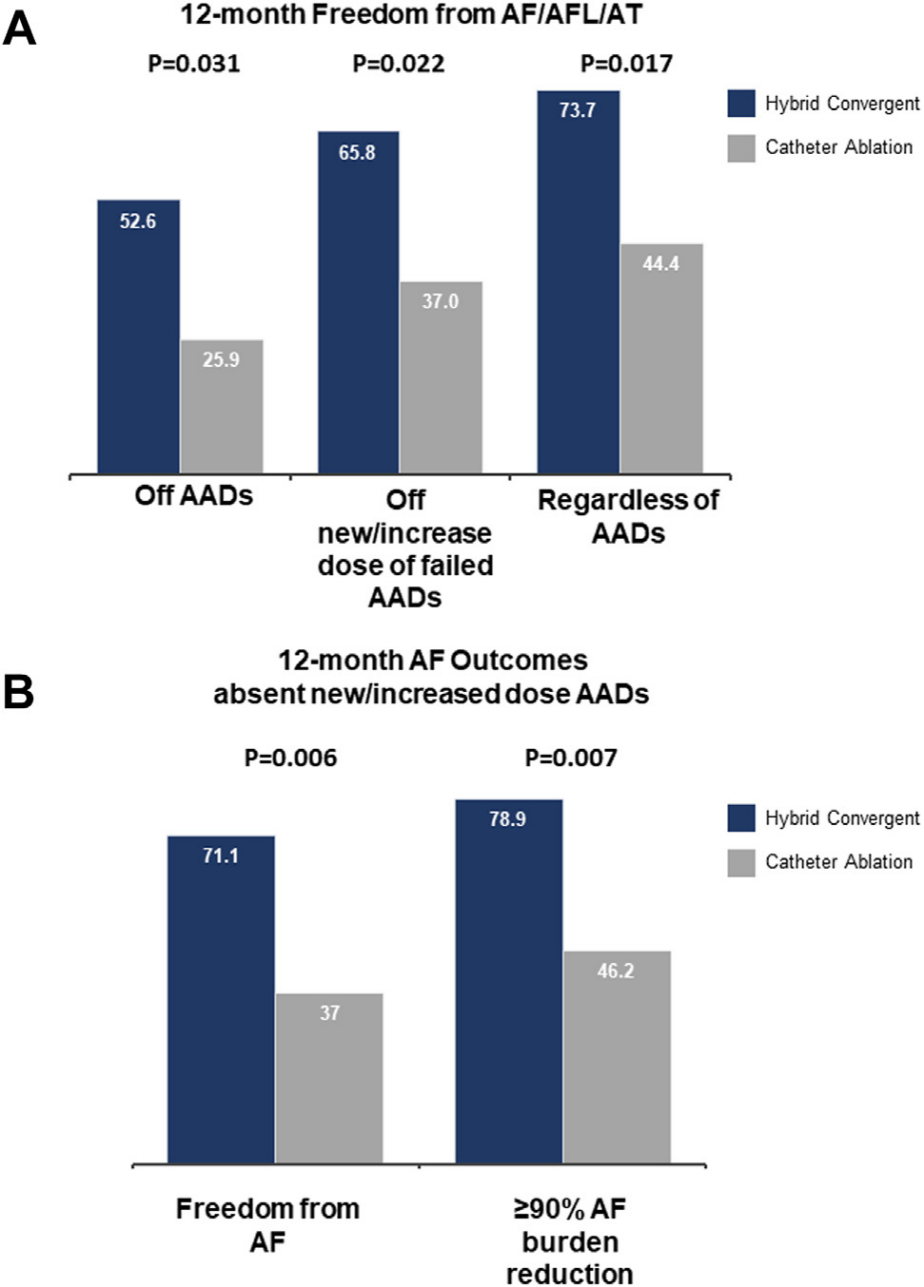
Freedom from atrial fibrillation (AF), atrial flutter (AFL), or atrial tachycardia (AT) and outcomes off new or increased dose antiarrhythmic drugs (AADs) at 12 months. *P* values were calculated for hybrid convergent vs catheter ablation using the chi-square test, as appropriate.

The primary effectiveness was freedom from AF, AFL, or AT without a new or increased dose of previously failed AAD through 12 months. Primary effectiveness was 65.8% (n = 25 of 38; 95% CI 50.7%–80.9%) in the hybrid convergent arm compared with 37.0% (n = 10 of 27; 95% CI 18.8%–55.3%) in the catheter ablation arm (Figure 1, Table 3). This was a 28.8% (95% CI 5.1%–52.4%) difference with improved effectiveness in favor of the hybrid convergent arm (*P =* .022). Freedom from AF, AFL, or AT regardless of AAD use through 12 months was 73.7% (n = 28 of 38; 95% CI 59.7%–87.7%) in the hybrid convergent arm compared with 44.4% (n = 12 of 27; 95% CI 25.7%–63.2%) in the catheter ablation arm (*P =* .017). In addition, the 12-month freedom from AF, AFL, or AT off class I or III AADs was 52.6% (n = 20 of 38; 95% CI 36.8%–68.5%) in the hybrid convergent arm compared with 25.9% (n = 7 of 27; 95% CI 9.4%–42.5%) in the catheter ablation arm (*P =* .031) (Figure 2, Table 3).

**Table 3:** Twelve- and 18-month effectiveness outcomes

Parameter	Hybrid convergent (n = 38)	Catheter ablation (n = 27)	Treatment difference	*P* value
Freedom from AF/AFL/AT off new AAD/increased dose previously failed AAD				
12 mo	65.8% (25/38), (50.7–80.9)	37.0 (10/27), (18.8–55.3)	28.8 (5.1–52.4)	.022
18 mo	60.5 (23/38), (50.0–76.1)	25.9 (7/27), (9.4–42.5)	34.6, (11.9–57.3)	.006
Freedom from AF/AFL/AT off AADs				
12 mo	52.6 (20/38), (36.8–68.5)	25.9 (7/27), (9.4–42.5)	26.7 (3.8–49.6)	.031
18 mo	47.4 (18/38), (31.5–63.2)	22.2 (6/27), (6.5–37.9)	25.2, (2.8–47.5)	.038
Freedom from AF/AFL/AT regardless of AADs				
12 mo	73.7 (28/38), (59.7–87.7)	44.4 (12/27), (25.7–63.2)	29.2 (5.8–52.6)	.017
18 mo	68.4 (26/38), (53.6–83.2)	33.3 (9/27), (15.6–51.1)	35.1 (12.0–58.2)	.005
≥90% AF burden reduction off new AAD/increased dose previously failed AAD				
12 mo	78.9 (30/38), (66.0–91.9)	46.2 (12/26), (27.0–65.3)	32.8 (9.7–55.9)	.007
18 mo	73 (27/37), (58.7–87.3)	36 (9/25), (17.2–54.8)	37.0, (13.3–60.6)	.004
Freedom from AF off new AAD/increased dose previously failed AAD				
12 mo	71.1 (27/38), (56.6–85.5)	37 (10/27), (18.8–55.3)	34.0 (10.8–57.3)	.006
18 mo	68.4 (26/38), (53.6–83.2)	29.6 (8/27), (12.4–46.9 )	38.8, (16.1–61.5)	.002

Values are % (n/n), (95% confidence interval) or % (95% confidence interval). *P* values calculated for hybrid convergent vs catheter ablation using chi-square test, as appropriate.

AAD = antiarrhythmic drug; AF = atrial fibrillation; AFL = atrial flutter: AT = atrial tachycardia.

Based on the 24-hour rhythm monitoring at 12 months, 78.9% (n = 30 of 38; 95% CI 66.0%–91.9%) of patients in the hybrid convergent arm had a ≥90% reduction in AF burden off new or increased dose of previously failed AAD compared with baseline vs 46.2% (n = 12 of 26; 95% CI 27.0%–65.3%) in the catheter ablation arm (*P =* .007) (Figure 2, Table 3). Freedom from AF without new or increased dose of AADs was 71.1% (n = 27 of 38; 95% CI 56.6%–85.5%) in the hybrid convergent group compared with 37.0% (n = 10 of 27; 95% CI 18.8%–55.3%) in the catheter ablation group (*P =* .006).

### Eighteen-month outcomes in LSPAF

**Figure 3:**
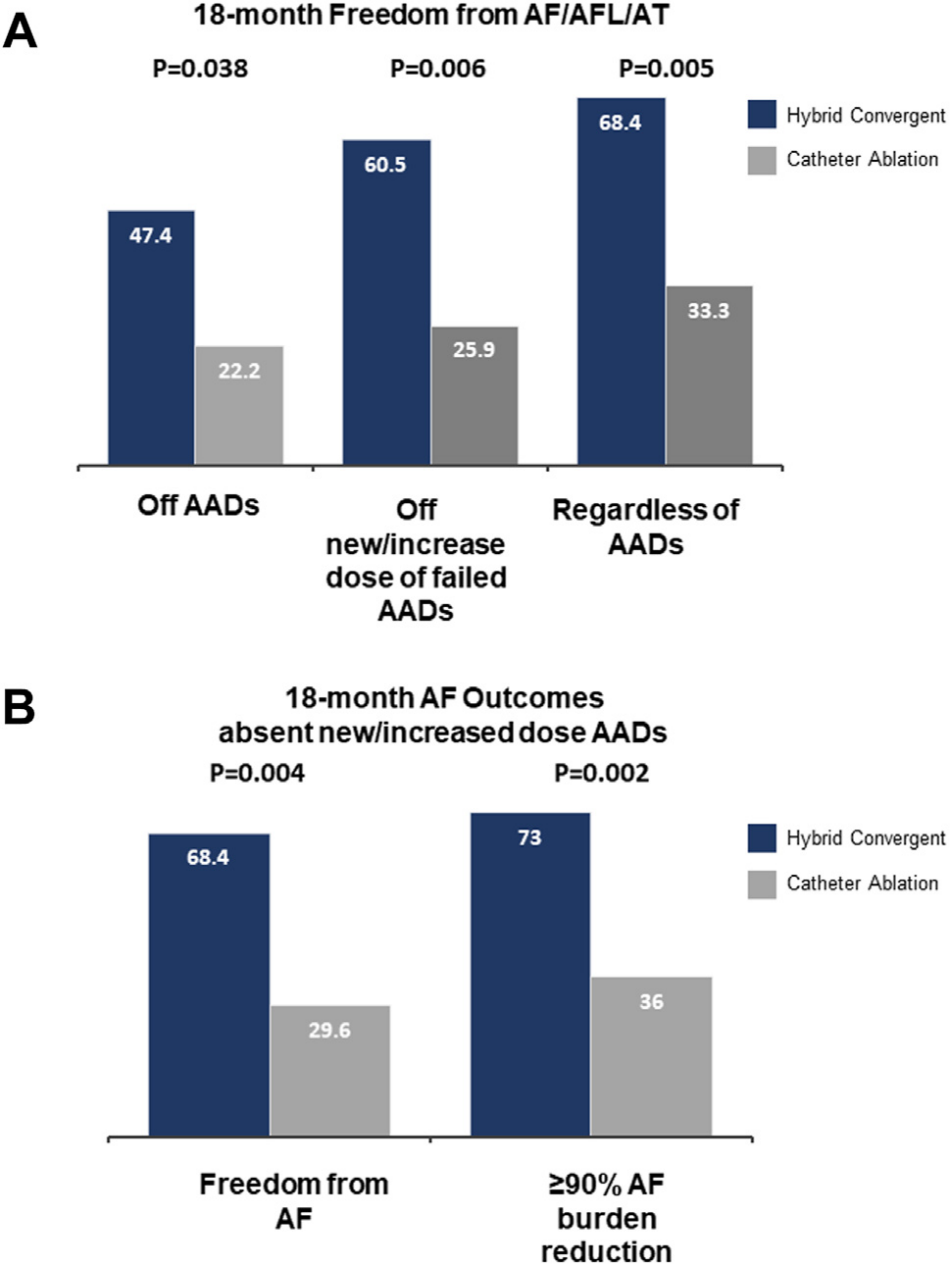
Freedom from atrial fibrillation (AF), atrial flutter (AFL), or atrial tachycardia (AT) **(A)** and outcomes off new or increased dose antiarrhythmic drugs (AAD) **(B)** at 18 months. *P* values were calculated for hybrid convergent vs catheter ablation using the chi-square test or Fisher’s exact test, as appropriate.

Effectiveness of hybrid convergent procedure was maintained through 18 months, with freedom from AF, AFL, or AT without a new or increased dose of previously failed AAD of 60.5% (n = 23 of 38; 95% CI 50.0%–76.1%) vs 25.9% (n = 7 of 27; 95% CI 9.4%–42.5%) in the catheter ablation arm (*P =* .006) (Figure 3, Table 3). Freedom from AF, AFL, or AT regardless of AADs through 18 months was 68.4% (n = 26 of 38; 95% CI 53.6%–83.2%) in the hybrid convergent arm vs 33.3% (n = 9 of 27; 95% CI 15.6%–51.1%) in the catheter ablation arm (*P =* .005). Off AADs, 18-month freedom from AF, AFL, or AT was 47.4% (n = 18 of 38; 95% CI 31.5%–63.2%) in the hybrid convergent arm compared with 22.2% (n = 6 of 27; 95% CI 6.5%–37.9%) in the catheter ablation arm (*P =* .038).

AF burden off new or increased dose of previously failed AADs at 18 months was evaluated by 7-day rhythm monitoring. In the hybrid convergent arm, 73% (n = 27 of 37; 95% CI 58.7%– 87.3%) of patients had ≥90% reduction in AF burden compared with 36% (n = 9 of 27; 95% CI 17.2%–54.8%) of patients in the catheter ablation arm (*P =* .004). Freedom from AF off new or increased dose of previously failed AADs through 18 months was 68.4% (26/38; 95% CI 53.6%–83.2%) in the hybrid convergent arm compared with 29.6% (n = 8 of 27; 95% CI 12.4%–46.9%) in the catheter ablation arm (*P =* .002).

### Repeat catheter ablations

Through 18 months postprocedure, there were 6 repeat catheter ablation procedures in 6 (15.8%) patients in the hybrid convergent arm for typical right atrial AFL (n = 3), atypical left atrial AFL (n = 1), AT (n = 1), and AF (n = 1). There were 5 repeat ablation procedures (2 catheter ablations and 3 hybrid convergent procedures) in 4 (14.8%) patients in the catheter ablation arm for AF (n = 4) (*P* = 1.000) (Supplemental Table 1).

### Safety in LSPAF

The primary safety endpoint was the incidence of predefined MAEs (procedural to 30-days postprocedure) in the hybrid convergent arm. In LSPAF patients, the MAE rate was 7.9% (n = 3 of 38; upper confidence limit 19.2%). This included 1 cardiac tamponade, 1 stroke, and 1 phrenic nerve injury. No deaths, atrioesophageal fistula, excessive bleeding requiring transfusion, transient ischemic attacks, severe PV stenosis, or myocardial infarctions occurred in the LSPAF patients. No MAEs occurred in the catheter ablation arm. No esophageal injuries were reported through 12 months postprocedure in either arm.

## Discussion

Completion of the prospective, multicenter randomized CONVERGE clinical trial marked more than a decade of research^11^ and refinement of procedural best practices.^12^ The trial results demonstrated superiority of hybrid convergent ablation compared with endocardial ablation for nonparoxysmal AF, with acceptable safety.^9^ One distinction of the CONVERGE trial compared with other contemporary ablation trials is the inclusion of patients with LSPAF (continuous AF duration for 1 year or more).^9^ Forty-two percent of patients in the CONVERGE trial had preprocedure LSPAF. Mean times since persistent AF diagnosis were 6.0 and 5.9 years in the hybrid convergent and catheter ablation arms, respectively. In comparison, mean durations of persistent AF were 0.6 years and 1.3 years in the STOP-PERSISTENT AF and PRECEPT (Safety and Effectiveness of STSF Catheter Evaluated for Treating Persistent Atrial Fibrillation) trials, respectively.^13^^,^^14^

Historically, LSPAF has been difficult to treat with traditional endocardial ablation strategies focused on PVI. The Hamburg study reported that 64.4% of patients with LSPAF experienced arrhythmia recurrence in the first year following a single endocardial ablation procedure.^15^ Long-term sinus rhythm maintenance was 20.3% after a single procedure and 45% after multiple procedures with ≥50 months median follow-up. Because non-PV triggers and substrate are likely more contributory to nonparoxysmal AF,^2^ durable ablation outside of the PVs may be important for favorable outcomes in LSPAF. However, a consensus endocardial extra-PVI ablation strategy that optimizes lesion durability, effectiveness, and safety has yet to be determined in a clinical trial for patients with LSPAF. Surgical ablation can be effective in LSPAF but is more invasive and primarily performed concomitantly with other planned cardiac surgery procedures.^5^ The combination of surgical and endocardial ablation in a minimally invasive stand-alone procedure may leverage advantages of both approaches for patients with LSPAF who do not require concomitant cardiac surgery. To further examine this possibility, we performed a post hoc analysis of LSPAF patients of the CONVERGE trial.

The results of this substudy showed that the rates of freedom from AF/AFL/AT regardless of AADs and off AADs were higher at 12 and 18 months compared with endocardial catheter ablation in the LSPAF population. A greater proportion of patients who received hybrid convergent ablation experienced ≥90% AF burden reduction off new or increased dose of AADs compared with those who received catheter ablation at 12 and 18 months. These results are notable among the reported single procedure success rates for endocardial ablation of LSPAF. As previously mentioned, Tilz and colleagues^15^ reported freedom from atrial arrhythmia recurrence of 35.6% at 12 months with endocardial catheter ablation for LSPAF. In the current substudy, 12-month freedom from atrial arrhythmia with or without AADs with hybrid convergent ablation was 74% but was 44% with catheter ablation.

Another distinction of the CONVERGE trial is that it was a randomized, controlled, 2-arm trial, thus permitting comparison of hybrid convergent ablation to conventional endocardial catheter ablation. Although the lesion set in endocardial catheter ablation arm did not include posterior wall isolation, there is currently no standardized approach to posterior wall isolation with endocardial catheters. Single-ring, box, and debulking strategies have been used. Endocardial posterior wall isolation is not a routine procedure and may pose additional safety risks, which should be weighed against mixed clinical outcomes reported in the clinical literature.^16^ Posterior wall reconnection rates have been reported to be approximately 40% after endocardial posterior wall isolation with RF,^17^^,^^18^ and endocardial cryoballoon posterior wall isolation often requires adjunctive RF for completion.^19^

A recent retrospective observational study reported a propensity score–matched comparison of catheter ablation vs hybrid convergent ablation for LSPAF, with mean duration of AF from 2.5 to 3 years.^20^ One procedural difference in this study compared with the CONVERGE trial was that the hybrid convergent procedure was staged with epicardial catheter ablation performed 6 weeks prior to the endocardial portion, rather than being performed in a single setting. Lesions outside the PVs in the control catheter ablation arm included a roof line in 23.3% or another ablation line 20.9% of cases, underscoring that endocardial posterior wall isolation was not a routine practice. The 12-month single procedure success rate on AADs was 60.5% with convergent hybrid ablation compared with 25.6% with catheter ablation (*P =* .002); there was also a significant difference off AADs (37.2% in hybrid convergent compared with 13.9% in catheter ablation; *P =* .025). The recent VENUS (Vein of Marshall Ethanol for Unablated Persistent AF) trial results suggest that even the addition of endocardial posterior wall isolation may not be able to achieve favorable clinical outcomes in advanced AF.^21^ In the VENUS trial, 52% of the patients in the catheter ablation–only arm had LSPAF and 75% of that arm received endocardial posterior wall isolation. The 12-month freedom from AF or AT off AADs was 38%. In the CONVERGE trial, 12-month freedom from AF, AFL, or AT off AADs was 52.6% and 65.8% off new AADs with hybrid convergent ablation in the LSPAF subgroup. While these trials are not a head-to-head comparison, the outcomes suggest that a hybrid epicardial-endocardial approach to posterior wall isolation may achieve higher clinical success than endocardial posterior wall isolation as a complement to PVI.

Improved outcomes with hybrid convergent ablation in LSPAF may be attributed to several factors. Ablating from both the epicardial and endocardial surfaces may help to overcome endocardial-epicardial dissociation, which has been observed in AF, including LSPAF.^22^ Aggregated evidence suggests that posterior wall isolation may be additive to PVI in persistent AF and LSPAF.^23^ The CONVERGE data suggest that if one can successfully or substantially ablate large contiguous portions of the posterior wall (in combination with PVI) that the results in LSPAF are significantly enhanced. Posterior wall box isolation with endocardial ablation differs from the epicardial posterior wall isolation in the hybrid convergent procedure. The hybrid convergent epicardial ablation can reach a lower part of the posterior wall compared with an endocardial box, which may result in autonomic ganglia ablation. The hybrid approach may be a consistent way of transmurally isolating the posterior wall with a low risk of thermal injury. While some atrioesophageal fitulas were reported in early iterations and experience with hybrid ablation,^24^^,^^25^ changes to the unipolar RF device, lesion set, and procedural steps have helped to mitigate this risk. No AEFs occurred in the CONVERGE trial.^9^ The 30-day MAE rate in the CONVERGE trial was 7.8% (n = 8 of 102). In the LSPAF subanalysis, the rate was 7.9% (n = 3 of 38), with 1 of the 3 MAEs, stroke, determined to be related to the endocardial ablation. In the CONVERGE trial, the most common MAE was delayed pericardial effusion that likely resulted from inflammation related to the pericardiotomy and ablation as opposed to cardiac perforation.^9^ These events were addressed by pericardial window. In 1 patient, there was delayed recognition of symptomatic pericardial effusion or tamponade leading to cardiac arrest. Subsequently, the patient had acute multiorgan dysfunction syndrome and anoxic brain injury. Best practices developed through the course of experience with the procedure suggest the use of pericardial drains, prophylactic anti-inflammatory medications, patient symptom monitoring and education, and a follow-up transthoracic echocardiogram or transesophageal echocardiogram within 1–3 weeks after the procedure to help mitigate pericardial effusion as a MAE.^12^

### Limitations

Limitations of this CONVERGE LSPAF subanalysis include the post hoc nature of this analysis and small population size of the subgroups. In addition, these data should be interpreted with caution because CIs and *P* values were not adjusted for multiplicity. Patients enrolled in the CONVERGE trial were randomized 2:1 to hybrid convergent and catheter ablation arms, but randomization was not stratified by baseline AF subtype. Patients in the catheter ablation arm did not receive posterior wall isolation, as discussed previously and previously as a limitation of the CONVERGE trial in the primary article.^9^ Although posterior wall isolation in the catheter ablation arm did not occur, complex fractionated atrial electrograms were allowed during CONVERGE at the investigator’s discretion and were performed in 18% of patients with LSPAF in the catheter ablation arm. Moreover, recent randomized trial data from the CAPLA (Catheter Ablation of the Left Atrial Posterior Wall in Persistent Atrial Fibrillation) trial suggest that endocardial posterior wall isolation is not additive to PVI.^26^

## Conclusion

This subanalysis of the CONVERGE clinical trial demonstrated that hybrid convergent ablation has improved effectiveness compared with catheter ablation in LSPAF with reasonable safety. This difference in effectiveness is maintained through at least 18 months. These results are important in the context of LSPAF, which has not been the focus of most clinical trials and for which no approved treatments existed. The data herein and other published real-world evidence recently resulted in Food and Drug Administration approval of this therapy for the treatment of symptomatic, drug-refractory LSPAF. Future studies should continue to evaluate outcomes in the LSPAF population and explore the role for hybrid convergent ablation as a second procedure in patients who have a prior failed left-sided ablation.

## Funding Sources

AtriCure, Inc was the sponsor of the CONVERGE trial.

## Disclosures

Dr DeLurgio has received honoraria, speaker fees, and is a consultant for AtriCure, Boston Scientific, and Medtronic; Dr Blauth is a consultant for New Cardioplegia Solutions and a proctor for AtriCure; Dr Halkos is on the advisory board and is a consultant for Medtronic; Dr Oza is a consultant for BioSense Webster and AtriCure; Dr Mostovych is a consultant for AtriCure; Dr Billakanty has received honoraria from AtriCure; Dr Ahsan has received speaker fees from AtriCure, Boston Scientific, and Johnson & Johnson; Dr Yap is a proctor for AtriCure; Dr Shults has received consultant fees and honoraria from Abbott Laboratories, AtriCure, and Medtronic; Dr Gilligan is a consultant for AtriCure; Dr Yang is on the advisory board and is a consultant for AtriCure; Dr Jacobowitz is a consultant for AtriCure; Dr Gill is a proctor for AtriCure. All other authors have reported that they do not have any conflicts relevant to the contents of this paper to disclose.

## Authorship

All authors attest they meet the current ICMJE criteria for authorship.

## Patient Consent

Patient informed consent form was obtained.

## Ethics Statement

The CONVERGE trial was conducted in accordance with the Declaration of Helsinki. Institutional Review Boards or ethics committee approval was obtained.
