# Supplementary material for: Hybrid epicardial-endocardial ablation for long-standing persistent atrial fibrillation: A subanalysis of the CONVERGE Trial
Source: Heart Rhythm O2. 2022 Dec 5;4(2):111–8. doi: 10.1016/j.hroo.2022.11.007 (PMC9975017; doi:10.1016/j.hroo.2022.11.007)
Supplement: Supplemental Table 1 [file mmc1.docx]

| **Supplemental Table 1. Ablative therapy (either catheter based or surgical ablation) received by study subjects within 18 months (+30 days) post-procedure ITT Population - LSPAF Patients** | | | | | |
| --- | --- | --- | --- | --- | --- |
| **Treatment Arm** | **Patient** | **Visit** | **Ablation Type** | **Ablation Procedure For** | **Ablation Locations** |
| Catheter Ablation | 1 | 18 month | Repeat Endocardial Catheter Ablation | AF | All 4 pulmonary veins and posterior wall of left atrium. |
|  | 2 | 12 month | Hybrid Convergent Ablation | AF | Left atrial appendage |
|  | 3 | 6 month | Hybrid Convergent Ablation | AF | surface of the left atrium and around pulmonary veins |
|  | 4 | 12 month | Hybrid Convergent Ablation | AF | 25 lesion sets were performed on the surface of the left atrium and around the right pulmonary veins. |
|  | 4 | 18 month | Repeat Endocardial Catheter Ablation | AF | anterior ridge and back wall |
| Hybrid Convergent | 1 | 12 month | Repeat Endocardial Catheter Ablation | Typical AFL (Right Atrial) | along the medial edge of scar line from posterior right atrium in a line between vena cava |
|  | 2 | 12 month | Repeat Endocardial Catheter Ablation | Atypical AFL (Left Atrial) | mitral block, re-isolation of the RIPV, Isolation of the posterior wall |
|  | 3 | 3 month | Repeat Endocardial Catheter Ablation | Typical AFL (Right Atrial) | typical atrial flutter line |
|  | 4 | 6 month | Repeat Endocardial Catheter Ablation | AF | right and left upper pulmonary veins plus CFAE mapping on anterior wall and roof |
|  | 5 | 6 month | Repeat Endocardial Catheter Ablation | AT | lower left side pulmonary vein lesion, left upper pulmonary vein anterior ridge and mitral isthmus line |
|  | 6 | Unscheduled | Repeat Endocardial Catheter Ablation | Typical AFL (Right Atrial) | CTI |
| AF=atrial fibrillation; AFL=atrial flutter; AT=atrial tachycardia; CFAE=complex fractionated atrial electrogram; CTI=cavotricuspid isthmus; LSPAF=longstanding persistent AF; ITT=intention to treat; RIPV=right inferior pulmonary vein. | | | | | |
